# Validating a Nutrition Ranking System for Food Pantries Using the Healthy Eating Index-2015

**DOI:** 10.3390/nu14193899

**Published:** 2022-09-21

**Authors:** Maria Fernanda Gombi-Vaca, Ran Xu, Marlene Schwartz, Michelle Battista Hesse, Katie Martin, Caitlin E. Caspi

**Affiliations:** 1 Rudd Center for Food Policy and Health, University of Connecticut, Hartford, CT 06103, USA; 2 Department of Allied Health Sciences, University of Connecticut, Storrs, CT 06269, USA; 3 Department of Human Development and Family Sciences, University of Connecticut, Storrs, CT 06269, USA; 4 Department of Health Professions, James Madison University, Harrisonburg, VA 22807, USA; 5 More Than Food Consulting, LLC, Avon, CT 06001, USA

**Keywords:** charitable food system, nutrition ranking, validity, Healthy Eating Index-2015

## Abstract

In 2020, charitable food organizations began adopting Healthy Eating Research (HER) nutrition guidelines, which rank individual foods in tiers (e.g., green, yellow, or red) based on each food’s nutrient profile. This study aimed to validate this HER tier-ranked system against the Healthy Eating Index-2015 (HEI) and develop a formula to summarize the percentages of tier-ranked foods in an overall nutritional quality index that correlated with HEI. Using secondary data of foods selected by clients in 16 Minnesota food pantries (*n* = 503 “client carts”), we generated an HEI score and percentages of green, yellow, and red foods for each cart. As validation, we tested the association between HEI scores and the percentages of tier-ranked foods and compared the means of the tier-ranked variables using quintiles of the HEI scores. HEI scores were positively associated with percentages of green foods and negatively associated with percentages of red foods. Next, we used statistical learning to generate weights to maximize the correlation of the tier-ranked variables and the HEI scores and used these weights to create an index. The index demonstrated a moderate-to-strong correlation with HEI and can be used as a single measure to summarize the overall nutritional quality for sets of tier-ranked foods.

## 1. Introduction

The charitable food system plays a critical role in providing food for households facing food insecurity [1,2,3]. Food insecurity is a household-level condition of limited or uncertain access to adequate food [4]. It is an indicator of economic instability, which is a core social determinant of health [5]. Due to the burdens of poor food access and social and economic barriers, the dietary patterns of those experiencing food insecurity are frequently suboptimal, with low intake of fruits and vegetables, dairy, and some micronutrients [6,7,8]. Those experiencing food insecurity are also at risk of diet-related chronic health conditions, including obesity and diabetes [9,10,11,12,13,14,15,16].

The charitable food system is composed of a network of food banks and food pantries. Typically, food banks are larger warehouses that procure and distribute food to food pantries within a specific geographic region. Most are part of large network of about 200 Feeding America food banks in the U.S. Food pantries are typically smaller, independent agencies who provide direct services. Those who visit food pantries—referred to as clients in much of the previous research literature [7,17,18,19,20,21,22], but also called neighbors or guests [23]—often visit food pantries frequently over a long period of time [17,18,24,25]. As a result, food from pantries may comprise a substantial portion of the total food for a household experiencing food insecurity [26]. In Minnesota, a statewide survey of food pantry clients indicated that the majority received half or more of their total food from the food pantry; visited the pantry at least monthly; and had been visiting for a year or more [26]. Growing evidence suggests that among those who rely on food pantries, food obtained at the food pantry has a higher nutritional quality than food from other sources [24,25,27,28]. This supports the notion that the charitable food system can play an important role in mitigating the diet-related effects of food insecurity by serving as a source of accessible, healthy food.

In recent years, charitable food agencies, including both food pantries and food banks, have increasingly devoted efforts to promote healthy food [1,2,29]. These efforts include implementing nutrition policies for their food inventory; offering nutrition education programs for their clients; improving the supply of healthy food within the system; and integrating behavioral economic strategies to promote healthy food selection at the food pantry [1,29,30,31,32].

System-level changes within the charitable food setting remain challenging. Food items are often shelf-stable and processed, containing high amounts of saturated fat, sodium, and added sugars [30]. There may be particular challenges in this sector in sourcing adequate fruit, dairy, and whole grains [33]. Many agencies also have limited staff and monetary resources and face other logistical challenges in healthy food distribution.

Moreover, it has been challenging to systematically measure and monitor the nutritional quality of food within the charitable food system. Until recently, efforts to establish and implement evidence-based nutritional standards within the charitable food system were fragmented. The gold-standard research measure of diet quality is the Healthy Eating Index (HEI), developed and evaluated by the National Cancer Institute (NCI) and the U.S. Department of Agriculture (USDA) [34,35]. This valid measure assesses the degree of alignment of a set of foods with the Dietary Guidelines for Americans (DGA) [36]. The HEI has been applied to the charitable food system [28,33,37]; however, the HEI calculation is difficult to conduct because it requires detailed nutrient information on each food product. The resources and logistics required to calculate the HEI make it impractical for widespread use in dynamic and low-resource settings like many charitable food agencies.

To address these challenges, alternate systems have been developed. For example, the Food Assortment Scoring Tool (FAST) is an index that correlates with the 2010 version of the HEI. The FAST was developed as an alternative measure that is calculated through a process of sorting and weighing food into 13 categories. However, its measurement still requires technical assistance and manual food classification and has not been widely adopted to date [38,39]. A 2017 survey among charitable food agencies showed many alternatives for nutrition tracking systems that were being implemented, including Feeding America’s Foods to Encourage (F2E) [40], Choosing Healthy Options Program (CHOP) Nutrient Analysis Tool [41], Supporting Wellness at Pantries (SWAP) [42], and others [31]. Although each of these systems align to some degree with the DGA, they use different nutrient criteria to categorize foods. Implementing common and practical guidelines in the charitable food system that align with the DGA is an important step in supporting the adoption of widespread practices to promote healthy food throughout the system—from procurement, to storage, to distribution. Adoption of a common set of guidelines can reduce the burden of nutrition tracking on managers, volunteers, and nutrition staff. It can also generate conversations about healthy food procurement with charitable food donors and can ultimately aid in decision-making around how agencies can best use their limited funds to procure healthy foods [30].

In 2019, Healthy Eating Research (HER), a national program of the Robert Wood Johnson Foundation, convened a panel of researchers and charitable food agency leaders to establish a set of evidence-based standards to be consistently applied in this food system. The “Healthy Eating Research Nutrition Guidelines for the Charitable Food System” (“HER Guidelines”) were released in March 2020 [30]. When developing the HER Guidelines, the expert panel referenced the existing SWAP nutrition ranking system [42] as a foundation upon which to build. The HER Guidelines offer a process for ranking individual food items into a three-tiered stoplight scheme (i.e., green (choose often), yellow (choose sometimes), red (choose rarely)) based on three key nutrients emphasized in the DGA: saturated fat, sodium, and added sugar. The HER Guidelines sort foods into 11 categories: (1) fruits and vegetables, (2) grains, (3) protein, (4) dairy, (5) non-dairy alternatives, (6) beverages, (7) mixed dishes, (8) processed and packaged snacks, (9) desserts, (10) condiments and cooking staples, and (11) other miscellaneous items. Each of the food categories have thresholds for the nutrients of concern. The HER Guidelines are meant to “support the availability of a more nutritious portfolio of food products across the charitable food system” [30] (p.17). These guidelines have since been endorsed by Feeding America, which released a toolkit to support implementation in 2021 [43]. While the use of the guidelines is not mandatory for charitable food agencies, with Feeding America’s endorsement, new systems have become available to make their adoption easier. For example, the SWAP system was revised in 2020 to align 100% with the new HER Guidelines and consists of a suite of tools to help food banks and pantries to implement the HER Guidelines. Other applications of the guidelines have evolved to align with these guidelines, such as Nourish [44].

Although the DGA informed the development of the HER Guidelines, the HER Guidelines have not been formally validated against measures of overall nutritional quality. This step is necessary because the HER Guidelines are used to rank individual foods but offer no guidance on measuring the total assortment of foods. The research to date uses the three values associated with the percentage by weight of foods in each tier (i.e., green, yellow, and red) to assess change over time, but this does not capture how changes in sets of foods (i.e., the mix of these three correlated variables) might demonstrate movement towards a more nutritious portfolio of food products in the charitable food system. For example, at this time, the relative impact of shifting red foods to yellow foods versus shifting yellow foods to green foods in improving nutritional quality is unknown. Understanding how changes in food assortment affects overall nutritional quality can support agencies in prioritizing the most impactful changes in food procurement and distribution.

To fill this gap, we examine a set of foods in the charitable food system that have been individually ranked according to HER Guidelines and scored with the HEI. The first aim of the study was to examine the validity of the HER Guidelines by using the HEI as the gold standard measure of the degree of alignment of an assortment of foods with the DGA. The second aim was to use statistical learning with this dataset to generate weights that could translate the percentage of green, yellow, and red foods into a single index that captures the overall nutritional quality score for any set of foods ranked using the HER Guidelines.

## 2. Materials and Methods

### 2.1. Data Source

We conducted a secondary analysis using data collected in 16 Minnesota food pantries (2018–2020) that included detailed nutrition information on all food items selected by clients at their food pantry visit (*n* = 503 “client carts”) [28].

In the primary study, a convenience sample of clients was recruited as part of an intervention evaluation (SuperShelf; https://www.supershelfmn.org/; accessed on 27 July 2022). The study took place in 16 choice-based Minnesota food pantries that were selected to participate in a multicomponent intervention to expand their food sourcing to focus on healthier food and use behavioral economics to make healthy choices easier for clients. The data include observations from both in the intervention and control conditions during the baseline and follow-up period. All clients were approached at the end of their visit during the data collection period at each food pantry and screened for eligibility. Eligible clients were at least 18 years old; had received food at the food pantry that day; had access to a phone; and spoke English, Spanish, or Somali. All participants signed informed consent documents and all procedures were conducted according to a human subject protocol approved by the University of Minnesota’s Institutional Review Board.

Research staff photographed the food each client selected at their visit and recorded the product name, brand, size, quantity, and special nutritional notes on the label (e.g., low sodium). Non-packaged items (e.g., fresh produce) were photographed on a scale with the weight displayed. Data from the photographs were first entered into an Excel database and then entered into Nutrition Data Systems for Research (NDSR). NDSR is a nutrient calculation software overseen by the University of Minnesota’s Nutrition Coordinating Center (NCC) that allows for direct and standardized entry of dietary data [45]. Its database includes over 18,000 foods, over 160,000 food variants, with values generated for 174 nutrients, nutrient ratios, and other food components. Foods were entered into NDSR using their exact profile or with the generic version. If there was no exact match or generic version of a food, either a substitute with a similar nutrient profile was entered or the food product was assembled as a recipe of individual ingredients using the nutrition facts label and ingredients list. Quality assurance procedures included a line-by-line review of records and consultation with a registered dietitian on entries flagged for review. The final dataset included the information needed to rank foods according to HER Guidelines as well as to calculate the Healthy Eating Index-2015.

### 2.2. HER Guideline Measures

Each food item in each of the 503 client carts (*n* = 21,030 items in total) was ranked according to the HER Guidelines. First, items were designated into 1 of 11 food categories (e.g., fruits and vegetables, dairy, desserts), and then assigned green, yellow, red, or not ranked based on the food category and amount of saturated fat, sodium, and added sugar per serving in the item. Detailed thresholds for each of these nutrients by food category are described elsewhere [30].

For this analysis, not-ranked foods (*n* = 1757) were excluded from the dataset. Not-ranked foods include condiments (e.g., salad dressing, soy sauce), cooking staples (e.g., flour, oil, sugar, baking soda), and miscellaneous foods (e.g., baby food). These foods are not scored using the HER Guidelines because they are not typically consumed on their own in large quantities, or they may be paired with more nutritious ingredients and used in the preparation of healthy meals. Miscellaneous foods are not ranked because they are intended for discrete populations with unique nutritional needs.

For each client cart, three tier-ranked variables were generated, representing the percentage by weight of green, yellow, and red foods. These variables were generated by dividing the amount in pounds of each of these categories of foods by the total amount in pounds of foods in the cart.

### 2.3. Healthy Eating Index (HEI) Measures

After excluding the not-ranked items, HEI scores were calculated for each client cart to measure its degree of alignment with the 2015–2020 Dietary Guidelines for Americans [46]. To calculate the 2015 version of the HEI scores, 28 ratios of dietary constituents to energy are derived and then scored for 13 subcomponents according to minimum and maximum standards [34]. The 13 subcomponents are summed to create a total score with a maximum score of 100. For the current analysis, only HEI total scores (not subcomponents) were used. For the validation process, we divided the sample of carts into quintiles according to their HEI score.

### 2.4. Data Analysis

Over the 503 client carts, the average percentage of foods by weight of green, yellow, and red and the average HEI score was calculated. We conducted a Pearson correlation analysis of HEI scores with each tier-ranked variable. Next, we compared the means of the tier-ranked variables using quintiles of HEI scores using linear regression models.

We then conducted statistical learning using the following approach. First, we selected two variables, the percentage of green foods and percentage of red foods, both of which demonstrated a statistically significantly association with HEI scores, to be included as model predictors with HEI score as the outcome variable. We fit three models: Model 1 using Ridge regression models, Model 2 using Ridge regression with cross-validation, and Model 3 using standard linear regression (ordinary least squares estimator), for comparison. Models 1 and 2 are statistical learning methods that use regularized regression to avoid overfitting the data and multicollinearity; these are used instead of linear regression models that may overfit the data. Ridge regression in Model 1 imposes a regularization penalty term based on information criteria, while the Model 2 uses cross-validation to optimize the penalization [47].

The coefficients obtained by each model were used as empirically generated weights for each tier-ranked variable to create a formula for an index that would best predict HEI scores. In other words, weights were generated to maximize the correlation of tier-ranked variables and HEI scores. The index scores were then calculated by summing the value of the estimated constant of the model and the weighted sum of the percentage of green and the percentage of red foods, where the coefficients for each tier-ranked variable generated by each model were used as the weights. Using the results from Models 1, 2, and 3, we generated scores for each Index A, B, and C, respectively, for each client cart in the sample. Each index was rescaled to have scores ranging between 0 and 100 to correspond with HEI.

To calculate the correlation of HEI with each of these indexes in a new sample, we conducted a 5-fold cross-validation analysis. First, we randomly divided our original client cart sample into 5 equivalent-sized samples, and designated them as Samples 1, 2, 3, 4, or 5. Then, we fit Model 1 (Ridge regression), Model 2 (cross validation), and Model 3 (linear regression) using Samples 2 to 5. The results obtained from these three models were used to calculate the scores for Indexes A, B, and C (using Ridge regression, cross validation, and linear regression, respectively) for Sample 1 (which was not included in model fitting). Next, we calculated the correlation between each Index and HEI for Sample 1. We repeated the same process (holding one sample, obtaining coefficients and constants to generate scores for all three indexes using the remaining samples, and then testing the correlation between indexes and HEI) for Samples 2, 3, 4, and 5.

For each Index A, B, and C, we calculated the average of their 5 correlation coefficients with HEI obtained from the 5-fold cross-validation on Samples 1 to 5. The average correlation represents the correlation between HEI and the Index A, B, or C in a new sample.

Finally, we selected the best fitting model (i.e., the model that generated the highest correlation between the index and HEI) and the entire original sample was transformed into a formula for the corresponding index that is a function of all three tier-ranked variables (percentages of green, yellow, and red), and that produces index scores that range from 0 to 100.

## 3. Results

Client cart HEI scores averaged 63.3 (SD = 11.4; ranged from 27.2 to 91.3); the percentage of green, yellow, and red foods averaged 50.1% (SD = 14.1), 26.0% (SD = 11.1), and 23.9% (SD = 12.4), respectively. The correlation between HEI score and the percentage of green foods was 0.522 (*p* < 0.001), while the correlation between HEI score and percentage of red foods was −0.531 (*p* < 0.001). The correlation between HEI score and the percentage of yellow foods was −0.066 (*p* = 0.137).

**Figure 1:**
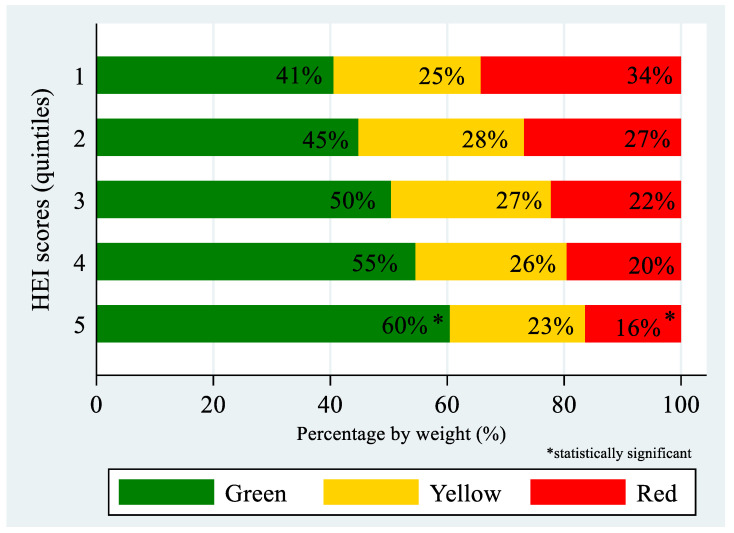
Percentage of green, yellow, and red foods by quintiles of HEI scores (*n* = 503).

Figure 1 shows the distribution of the three tier-ranked variables as a function of HEI quintiles. Higher quintiles of HEI scores were associated with higher percentages of green foods and lower percentages of red foods (*p*-value for trend < 0.001). The association between quintiles of HEI scores with percentage of yellow foods did not reach statistical significance (*p*-value for trend = 0.058).

The statistical learning results from the Models 1, 2, and 3 are reported in Table 1. The coefficients and constants from the models were used to create a formula to calculate scores for Indexes A, B, and C. The average score for Index A was 69.0 (SD = 13.6), for Index B was 69.0 (SD = 13.6), and for Index C was 68.7 (SD = 13.7).

**Figure 2:**
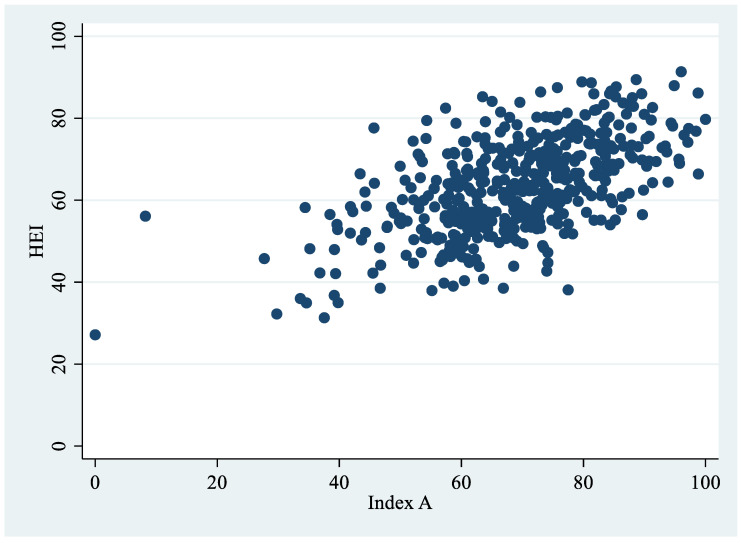
Correlation between HEI and Index A (*n* = 503).

The correlation coefficients obtained using the 5-fold cross-validation are found in Table 2. The correlations between HEI and Indexes A, B, and C were very similar between all three methods (Ridge regression, cross-validation, and linear regression, respectively), but correlations for Index A generated by Ridge regression (Model 1) were slightly higher. The average correlation of HEI with Index A in a new sample was estimated to be 0.585 (SD = 0.06; *p* < 0.001), with Index B to be 0.585 (SD = 0.06, *p* < 0.001), and with Index C to be 0.584 (SD = 0.06, *p* < 0.001). Figure 2 shows the correlation between HEI and Index A in the sample analyzed.

Using the results obtained from Model 1, we transformed the formula for Index A into a function of all three tier-ranked variables, as follows:

(1) Index=((((0.7773×Green)+(0.5923×Yellow)+(0.3753×Red))−37.53)/40.20)×100

This formula for the index produces scores that range from 0 to 100. A set of foods that is 100% green will have an index score of 100, a set of foods that is 100% red will have a score of 0, and a set of foods that is 100% yellow will have a score of 54.

## 4. Discussion

This study found an expected relationship between HEI scores and tier-ranked variables (percent of red, yellow, and green foods) for sets of foods ranked using the HER system. It also found a moderate-to-strong correlation between the HEI and the Index A summarizing the three values from a set of foods based on the HER Guidelines used in the charitable food system. The summary Index A, which we name the “Charitable Food Nutrition Index (CFNI)”, offers a simple formula to calculate a score of overall nutritional quality for any set of foods.

Each of the tier-ranked variables was associated with HEI scores in the hypothesized direction: the percentage of green foods was significantly positively correlated; the percentage of red foods was significantly negatively correlated; and the percentage of yellow foods was not significantly correlated with HEI. The findings for the green and red percentages were not surprising because the criteria used to rank foods and the HEI scoring system are both based on the DGA. The finding that the percentage of yellow foods is not independently related to HEI is also unsurprising, as it is not possible to interpret nutrition quality based on the percentage of yellow foods alone. In other words, as an outcome measure, changes in the yellow category are only interpretable when combined with information on how the proportions changed. A shift from yellow to red will result in a negative shift and from yellow to green will result in an improvement.

The correlation of 0.585 between HEI and the CFNI based on a tier-ranked set of foods is considered a moderate-to-strong correlation [48,49]. A correlation of approximately this magnitude was expected given that: (a) only two variables were used as predictors, (b) HEI is based on nutrient density per 1000 calories while the tier-ranked variables are based on measurement of nutrition quality per unit weight, and (c) tier-ranked measures use only three nutrients to obtain a rank, while the 2015 version of HEI scores is based on 13 subcomponents. This correlation is slightly lower than the correlation between the 2010 version of the HEI and the FAST found in a previous study, which was 0.66 for client carts [39]. Similar to the FAST, the CFNI is derived by multiplying the proportion of food in different categories by a coefficient that maximizes the correlation with the HEI and summing the categories. The FAST requires sorting and weighing food in 13 categories; while largely based on Food Bank Codes (FBCs), the standard classification system used by Feeding America network agencies, FAST categories do not perfectly align with FBC categories and require some technical support to implement. Meanwhile, Feeding America has begun providing substantial financial supports to food banks to help incentivize the use of the HER Guidelines; along with the availability of technical support for implementing the guidelines [43], adoption of guideline-based ranking systems has been growing (CT Foodshare, personal communication, 21 December 2021). A next step is better integrating HER Guideline food categories into agency inventory tracking platforms.

The CFNI that resulted from these analyses can potentially benefit both researchers and charitable food agencies in a variety of ways to measure the healthfulness of inventory in the charitable food system. For researchers, the CFNI provides a single, continuous measure of nutritional quality that aligns with the DGA. The research on stoplight systems has required separate analyses for changes in green and red proportions, which leaves the question of how to compare the importance of an increase in green or a decrease in red. By consolidating these values into a single score associated with the HEI, there can be a streamlined and meaningful statistical test of the impact of an intervention to improve the nutrition landscape within a food pantry or food bank.

There is also potential for this single value to be used by food banks and other agencies, alone or in conjunction with the raw percentages of green, yellow, and red foods. For example, a single measure provides a way to monitor changes in the nutritional quality of food over time; to understand differences in nutritional quality across food procurement streams (e.g., federal programs, food purchases, and donations); and to report improvements in nutritional quality to funders, donors, or other stakeholders. Alternately, the CFNI can be added to food-ordering systems to provide feedback on the nutritional quality of food ordered in real time. Indeed, evidence suggests that when food pantries receive nutritional quality information when ordering from food banks, they select healthier food [50,51]. Communication with food banks using SWAP suggests that some currently gauge their nutritional quality score by adding the green and yellow percentages together. This eliminates the nutritional distinction between green and yellow foods and may result in a “ceiling effect” where, after minimizing red foods, agencies can no longer observe increases. The CFNI measure allows for a score improvement as a greater proportion of inventory moves from yellow to green.

Notably, food pantry practices and processes have been upended in many ways by the COVID-19 pandemic. This period was characterized by challenges in the food supply chain and an initial surge in food distribution [52], followed by periods of uneven demand for charitable food as various COVID-19 relief measures were implemented and discontinued. In some cases, systems for tracking and providing healthy food were suspended and abandoned as agencies focused on simply moving food through the system [53]. As new phases of the pandemic continue, it remains to be seen how changes in nutrition tracking in food pantries will be prioritized or implemented by agencies.

A strength of this study was the use of statistical learning techniques to obtain an index that maximizes the correlation with a rigorous measure of diet quality that measures alignment with the DGA. We tested two different methods of statistical learning and compared them with standard linear regression to obtain the weights for the tier-ranked variables that best predicted HEI score. The CFNI is based on the HER Guidelines, a system that is being increasingly adopted in food banks and pantries and is endorsed by Feeding America.

A limitation of this study is that it is based on data from 16 food pantries in one U.S. state, and that these food pantries, selected to participate in an intervention, may have healthier food, on average, than many food pantries; however, the red, yellow, and green foods that comprise this dataset are likely similar to foods found in pantries in other areas of the country. Additionally, HEI scores in the dataset spanned a wide range, between 27 and 91. A practical limitation of this work is that the creation of another measure may be overwhelming or seem unnecessary to agencies if they are accustomed to a three-tiered ranking system. More research is necessary to determine how the CFNI is best interpreted, and how sensitive the index scores are to changes that result from interventions in the charitable food system.

## 5. Conclusions

The results of this study establish the validity of the HER Guidelines compared with the HEI, the gold standard measure of overall nutrition quality based on DGA. HER Guidelines correlated with the HEI when they are used as tier-ranked variables and when they are used as an index. This validation is essential as the implementation of these guidelines is beginning to occur on a widespread level across the charitable food system. Moreover, the CFNI based on HER Guidelines generated in this study is a tool that can be used to summarize the overall nutritional quality for any set of tier-ranked foods. The CFNI correlates well with HEI scores and is calculated using a simple formula that can be easily integrated into ordering and inventory systems, and can be used to track nutrition quality, to generate reports for sponsors and agencies, and to evaluate the results from academic studies and interventions. Measuring nutritional quality in the charitable food system presents challenges. Validated measures, when paired with toolkits designed to put recommendations into practice, can ultimately be used to provide more nutritious food when improved food security, diet quality, and diet-related health outcomes are the priority.

## Figures and Tables

**Table 1:** Coefficients from the statistical learning methods using Ridge regression (Model 1), cross-validation (Model 2), and linear regression (Model 3), using percentage of green foods and percentage of red foods as predictors and HEI score as the outcome (*n* = 503).

Tier-Ranked Variables	Model 1 (Ridge Regression)	Model 2 (Cross-Validation)	Model 3 (Linear Regression)
% Green	0.185	0.257	0.270
% Red	−0.217	−0.269	−0.278
Constant	59.23	57.13	56.72

**Table 2:** Pearson’s correlation coefficients between HEI and tier-ranked index scores obtained using 5-fold cross-validation analysis.

Sample	Sample Size	Index A	Index B	Index C
1	101	0.4863	0.4865	0.4866
2	100	0.5998	0.5997	0.5997
3	101	0.6452	0.6444	0.6441
4	100	0.5641	0.5642	0.5642
5	101	0.6274	0.6276	0.6276
*Mean*		0.5846	0.5845	0.5844
*SD*		0.0629	0.0626	0.0625

Index A, using Ridge regression; Index B, using cross-validation; Index C, using linear regression; all correlations were statistically significant (*p* < 0.05).

## Data Availability

Raw data will be retained and is readily available if requested.
